# Monitoring of Cerebral Metabolism in Postcardiac Arrest Patients: A Pilot Study

**DOI:** 10.1089/ther.2018.0018

**Published:** 2018-12-05

**Authors:** Minjung Kathy Chae, Sung Eun Lee, So Young Kang, Min Seob Sim

**Affiliations:** ^1^ Department of Emergency Medicine, Ajou University School of Medicine, Suwon, Korea.; ^2^ Department of Neurology, Ajou University School of Medicine, Suwon, Korea.; ^3^ Office of Biostatistics, Institute of Medical Sciences, Ajou University School of Medicine, Suwon, Korea.; ^4^ Department of Emergency Medicine, Samsung Medical Center, Sungkyunkwan University School of Medicine, Seoul, Korea.

**Keywords:** heart arrest, brain injuries, energy metabolism

## Abstract

For better care of postcardiac arrest patients, objective serial assessments of brain injury severity are needed. We hypothesized that monitoring of cerebral energy metabolism based on arterio-jugular (AJ) differences of metabolites will provide serial details of brain injury and information about neurologic outcomes in patients. Measurements of lactate and glucose in addition to blood gas analyses were done every 6 hours from the radial artery and jugular bulb in postcardiac arrest patients throughout targeted temperature management (TTM). Jugular bulb saturation, AJ difference of O2, and AJ difference of lactate (AJDL) were calculated and compared between the different neurologic outcome groups. Linear mixed-model analysis was done to assess AJDL based on the different phases of TTM and neurologic outcome. A total of 13 patients were included in the study (*n* = 4 good outcome, *n* = 9 poor outcome). AJDL as an indicator of cerebral metabolism was significantly different between the outcome groups and demonstrated negative values in the poor neurologic outcome group (0.06 [0.05–0.09] vs. −0.14 [−0.06 to −0.27], *p* < 0.01). However, there was no significant difference in AJDL between the outcome groups in the mixed effects model (*p* = 0.05). In addition, there were no differences between the phases of TTM in both groups (*p* = 0.46). AJDL was observed to be informative but was not significantly different between neurologic outcome groups throughout the different phases of TTM in our pilot study. Future studies are needed for further investigation of AJDL as an indicator of brain injury severity.

## Introduction

Cardiac arrest survivors have high mortality and poor neurologic prognosis due to global hypoxic-reperfusion brain injury (Callaway *et al.*, 2015). Understanding the pathophysiologic mechanism of the ongoing injury cascade is of clinical importance in these patients (Yenari and Han, 2012). Furthermore, for patient specific interventions such as tailored targeted temperature management (TTM) for postcardiac arrest patients, objective and serial assessments of the severity of brain injury are needed.

Global cerebral oxygenation and metabolism can be monitored by blood gas analysis and arterio-jugular (AJ) differences of metabolites from the artery and jugular bulb and have been used in other etiologies of brain injury (Perez *et al.*, 2003; Leyvi *et al.*, 2006; Jalloh *et al.*, 2013; Tholance *et al.*, 2015). The AJ difference of lactate (AJDL) reflects the net uptake or export of lactate from the brain and provides information about the severity of brain damage in traumatic brain injury and subarachnoid hemorrhage (Jalloh *et al.*, 2013; Tholance *et al.*, 2015). However, there are insufficient studies on these parameters in postcardiac arrest patients.

Therefore, we examined the hypothesis that cerebral metabolic indicators, including AJDL levels, would be different between different neurologic outcomes in postcardiac arrest patients.

## Methods

Samsung Medical Center Institutional Review Board (2015-09-007-003) approved this study. As this was a retrospective observational study, patient consent forms were waived.

### Study design and population

This is a retrospective single center observational study of resuscitated cardiac arrest patients over 18 years of age admitted after out-of-hospital cardiac arrest for postcardiac arrest care and TTM with a jugular bulb catheter and arterial line insertion. The treating hospital is an urban tertiary care university hospital in Seoul, Korea, and the study period was from April 2014 to May of 2015. After admission to the intensive care unit, sono guided jugular bulb catheter placement was performed for monitoring of cerebral oxygenation and metabolism for detecting possible secondary brain injury. Patients with a “do not resuscitate” request from families or patients with withdrawal of treatment, extracorporeal membrane oxygenation applied patients, patients with a severe bleeding tendency, or with other conditions with increased risk of complications of jugular bulb catheter insertion were excluded.

### Targeted temperature management

All patients were treated following a standardized TTM protocol. TTM was conducted with cooling pads with a feedback loop (Arctic Sun^®^ Energy Transfer Pads™; Medivance Corp., Louisville). The target temperature of 33°C was maintained for 24 hours with rewarming to 36.5°C at a rewarming rate of 0.15°C/h, followed by normothermia for another 24 hours. Mandatory sedation and analgesics were provided, and control over events of seizure and shivering was done when required, based on the protocol.

### Jugular bulb catheter insertion

Jugular bulb catheter was placed by sonography guided retrograde cannulation in the dominant jugular vein. Correct position was confirmed by lateral neck x-ray to check that the tip of the catheter was placed above the lower border of the first cervical spine (C1). After correct placement, heparin lock was done for adequate patency. Blood gas analyses and measurement of lactate and glucose were done every 6 hours from the radial artery and jugular bulb throughout the different phases of TTM. The blood gas analyses were analyzed by alpha stat.

### Calculated measurements of cerebral oxygenation and metabolism from arterial and jugular laboratory results

Jugular bulb saturation (SJO2) is considered to reflect the brain's oxygen demand and supply, with a normal range of 55–75%. Low levels of SJO2 may indicate high oxygen demand exceeding supply, while higher levels may indicate high oxygen supply or low cerebral demand, sometimes caused by profound injury and inability to use oxygen (Buunk *et al.*, 1999). The AJ difference of O2 (AJDO2), calculated by subtracting SJO2 from arterial oxygen saturation (SaO2), also is thought to provide information of cerebral metabolic demand, with higher levels indicating high oxygen demand and lower levels indicating low oxygen demand, possibly due to severe injury with mitochondrial damage (Andrews *et al.*, 1991). AJDL is calculated by subtracting jugular lactate from arterial lactate and provides an outline of net uptake of export of lactate from the brain. Lactate, generally conceived as an anaerobic waste product, is an important energy source for the brain, especially after brain injury resulting in lactate uptake (Schurr *et al.*, 1997; Bartnik *et al.*, 2005; Bouzat and Oddo, 2014). However, during extensive brain damage with mitochondrial dysfunction or anaerobic metabolism, the brain may have lactate production exceeding consumption leading to lactate export (Cruz *et al.*, 1993). Negative AJDL has been suggested to imply severe brain damage in other etiologies of brain injury (Cruz *et al.*, 1993; Perez *et al.*, 2003; Chieregato *et al.*, 2007; Tholance *et al.*, 2015).

The calculated measurements were evaluated during patient treatment, and under instances of derangements outside of the normal ranges or drastic changes (Schurr *et al.*, 1997; Chieregato *et al.*, 2007), hemodynamic parameters and oxygenation were reviewed to see if there were any reversible causes of suspected secondary brain injury.

### Data collection

We retrospectively reviewed data of the enrolled patients from a prospectively documented TTM database, including demographic data, cardiopulmonary resuscitation (CPR) data, TTM data, and neurologic outcomes expressed as the Glasgow–Pittsburgh Cerebral Performance Category (CPC) at 1 month after return of spontaneous circulation (ROSC). CPC was dichotomized into good neurologic outcome (CPC 1-2) and poor neurologic outcome (CPC 3-5). CPR data included witnessed arrest, bystander CPR, initial rhythm, presumed cause of arrest, time from collapse to CPR that was defined as no flow time, and time from CPR to ROSC that was defined as low flow time. Arterial and jugular bulb laboratory results along with the calculations were documented in the electronic medical records (EMR) from the treating physician. Further information considering different phases of TTM and time of the arterial and jugular measurements were also achieved from the EMR.

### Statistical analysis

Continuous variables were reported as median and interquartile range (IQR), and Wilcoxon's rank sum test was conducted for comparison. Linear mixed model analysis was conducted to assess AJDL based on the different phases of TTM and with respect to neurologic outcome. Data were analyzed using Stata software version 14 (Stata Corp., LP, TX) and SAS version 9.4 (SAS Institute, Cary, NC).

## Results

**FIG. 1.**
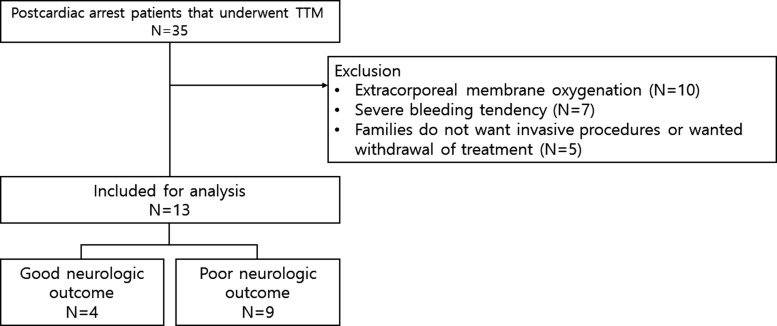
Study flow of TTM. TTM, targeted temperature management.

A total of 13 patients were included for analysis, and 4 patients demonstrated good neurologic outcome while 9 patients demonstrated poor neurologic outcome (Fig. 1). A total of 168 arterial jugular blood gas analyses (*n* = 53 good, *n* = 115 bad), 165 arterial jugular lactate samples (*n* = 53 good, *n* = 112 poor), and 166 arterial jugular glucose samples (*n* = 53 good, *n* = 113 poor) were obtained. The demographic characteristics of the 13 patients are shown in Table 1.

**Table 1.** Patient Characteristics

*No.*	*Age*	*Sex*	*Cause of arrest*	*AED shock*	*No flow time*	*Low flow time*	*CPC*
1	41	F	Asphyxia	No	Unknown	25	5
2	26	F	Cardiogenic	Yes	Unknown	Unknown	2
3	67	M	Asphyxia	Unknown	0	15	5
4	51	M	Cardiogenic	No	17	6	5
5	69	M	Unknown	No	Unknown	29	5
6	85	F	Asphyxia	No	Unknown	15	4
7	52	M	Cardiogenic	Unknown	5	13	1
8	36	M	Cardiogenic	Yes	9	5	1
9	50	M	Unknown	Yes	6	24	5
10	55	M	Unknown	No	Unknown	Unknown	4
11	29	F	Cardiogenic	No	20	25	5
12	27	M	Cardiogenic	No	2	69	5
13	71	M	Cardiogenic	Yes	0	8	1

AED, automated external defibrillator; CPC, cerebral performance category.

### Comparison of cerebral metabolism indicators between neurologic outcome groups

AJDL was significantly different between both neurologic outcome groups. While patients with good neurologic outcome had positive AJDL indicating lactate uptake, patients with poor neurologic outcome had negative AJDL, indicating lactate export (0.06 [0.05–0.09] vs. −0.14 [−0.06 to −0.27], *p* < 0.01). SJO2 was higher in the poor neurologic outcome group (76.0 [73.6–80.1] vs. 83.9 [75.9–87.8], *p* = 0.32) but was not statistically significant. Although AJDO2 seemed to be higher in the good neurologic outcome group, it was not statistically significant (17.1 [15.1–18.9] vs. 11.5 [8.6–17.4], *p* = 0.25). Arterio-jugular difference of glucose was also higher in the good neurologic outcome group without significance (5.5 [4.5–6.3] vs. 3 [3–4], *p* = 0.05). Trends of these metabolic indicators over phase of TTM are presented in Table 2.

**Table 2.** Values of Cerebral Metabolism Indicators

*Phase*	*Hypothermia*	*Rewarming*	*Normothermia*	*Post TTM*	*Total*	p
SJO2						
Good outcome	71 (68.8 to 81.6)	84 (81.1 to 86.9)	75.7 (72.9 to 81.7)	76.7 (65.2 to 81.3)	76.0 (73.6 to 80.1)	0.32
Poor outcome	91.5 (88.2 to 95.3)	85 (74.0 to 89.8)	82.6 (73.2 to 88.1)	84.8 (81.0 to 86.9)	83.9 (75.9 to 87.8)	
AJDO2						
Good outcome	18.1 (15.3 to 23.4)	10.2 (10.0 to 10.4)	16.8 (14.3 to 17.6)	18.2 (16.1 to 31.9)	17.1 (15.1 to 18.9)	0.25
Poor outcome	6.6 (2.9 to 7.3)	11.0 (7.4 to 14.0)	14.2 (7.9 to 20.9)	11.0 (10.8 to 14.1)	11.5 (8.6 to 17.4)	
AJDL						
Good outcome	0.11 (−0.02 to 0.12)	0.04 (0.02 to 0.1)	0.04 (0.03 to 0.09)	0.01 (−0.05 to 0.07)	0.06 (0.05 to 0.09)	<0.01
Poor outcome	−0.05 (−0.24 to 0.15)	−0.1 (−0.25 to −0.05)	−0.09 (−0.3 to −0.05)	−0.14 (−0.28 to −0.12)	−0.14 (−0.06 to −0.27)	
AJDglc						
Good outcome	5.5 (0 to 7)	3.25 (3.0 to 3.5)	6.0 (5.0 to 8.8)	4.0 (2.5 to 5.0)	5.5 (4.5 to 6.3)	0.05
Poor outcome	6.0 (−1 to 6)	3 (2.0 to 3.0)	4.5 (4.0 to 6.0)	2.5 (2.0 to 4.0)	3 (3.0 to 4.0)	
AJDCO2						
Good outcome	−7.7 (−8.3 to −7.1)	−4.3 (−6.6 to −2.0)	−6.5 (−7.1 to −5.4)	−6.0 (−8.1 to −4.0)	−7.0 (−5.6 to 7.1)	0.22
Poor outcome	−4.6 (−5 to −4.6)	−5.0 (−6.2 to −3.2)	−6.0 (−6.3 to −4.0)	−6.1 (−6.1 to −5.9)	−5.5 (−4.3 to 6.1)	

Data are expressed as median and quartiles. *p*-Value is from comparison of metabolism indicators during the total phase of TTM between outcome groups.

AJDCO2, arterio-jugular difference of CO2; AJDglc, arterio-jugular difference of glucose; AJDL, arterio-jugular difference of lactate; AJDO2, arterio-jugular difference of O2; SJO2, jugular bulb saturation; TTM, targeted temperature management.

### Serial AJDL based on different phases of TTM with respect to neurologic outcome

**FIG. 2.**
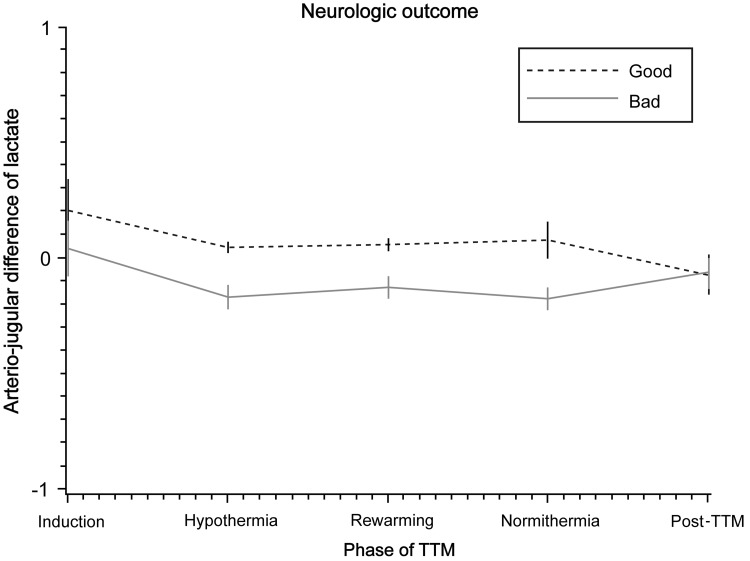
Mixed effects model of AJDL by TTM phase and neurologic outcome. There was no interaction between neurologic outcome and hypothermia phase (*p* = 0.80). There was no significant difference between the phases of TTM (*p* = 0.46). There was no significant difference in AJDL between outcome groups (*p* = 0.05). AJDL, arterio-jugular difference of lactate; TTM, targeted temperature management.

We further assessed serial AJDL based on different TTM phase and with respect to neurologic outcome (Fig. 2). However, there was no significant difference in AJDL between the outcome groups in the mixed effects model (*p* = 0.05). In addition, there were no differences between the phases of TTM (*p* = 0.46).

## Discussion

Previous studies focusing on AJDL in postcardiac arrest patients are limited. In our pilot study, AJDL was different between the outcome groups involving postcardiac arrest patients, with negative AJDL implying lactate export in patients with poor neurologic outcome. These findings were apparent even during the early phases of TTM and could be used as a prognostic marker earlier in resuscitation. Jugular bulb catheter insertion was achieved with safety and minimal invasiveness with no complications in the 13 cases.

Lactate is considered as an important energy source for the injured brain, and previous studies have reported that lactate generated by astrocytes is shuttled to neurons as oxidative fuel. However, under conditions of extensive brain damage associated with mitochondrial dysfunction, anaerobic metabolism with lactate production exceeding consumption can result in cerebral lactate export or negative AJDL (Dienel, 2014; Schurr, 2014).

Other findings of our study included higher SJO2 and lower AJDO2 in the poor outcome group. Although these findings were not statistically significant, the application of higher SJO2 (Buunk *et al.*, 1999) and lower AJDO2 as an indicator of irreversible brain damage or bad outcome was found to be similar to the results of previous studies.

This study has several limitations. First, as the study was a retrospective study, the results of this study may not be generalized, and any medical interventions that might have affected the variables over time were not considered. Second, as the number of patients in the study was limited and from a single center, larger prospective studies with more patients will be needed to confirm the results of this study. Third, considering the fact that the cerebral blood flow can affect the assessed parameters, this particular aspect was not considered in the analysis. Nevertheless, as a minimally invasive and safe method, serial AJDL measurements showed potential to provide objective information about the severity of brain injury.

## Conclusions

AJDL seemed to provide serial information regarding brain injury severity throughout the different phases of TTM but exhibited no significance in our pilot study conducted on a limited sample volume. Future prospective studies and further validation of AJDL may open potential for tailored temperature management by brain injury severity, rather than a “one fits all” approach of TTM in cardiac arrest survivors.
